# Identification of Copy Number Variants in a Southern Chinese Cohort of Patients with Congenital Scoliosis

**DOI:** 10.3390/genes12081213

**Published:** 2021-08-05

**Authors:** Wenjing Lai, Xin Feng, Ming Yue, Prudence W. H. Cheung, Vanessa N. T. Choi, You-Qiang Song, Keith D. K. Luk, Jason Pui Yin Cheung, Bo Gao

**Affiliations:** 1 School of Biomedical Sciences, Li Ka Shing Faculty of Medicine, The University of Hong Kong, Hong Kong, China; floralai@connect.hku.hk (W.L.); FengxinCassie@connect.hku.hk (X.F.); nervym7@connect.hku.hk (M.Y.); vntchoi@hku.hk (V.N.T.C.); songy@hku.hk (Y.-Q.S.); 2 Department of Orthopaedics and Traumatology, The University of Hong Kong, Hong Kong, China; gnuehcp6@hotmail.com (P.W.H.C.); hrmoldk@hku.hk (K.D.K.L.)

**Keywords:** congenital scoliosis, congenital vertebral malformation, copy number variant, CNV

## Abstract

Congenital scoliosis (CS) is a lateral curvature of the spine resulting from congenital vertebral malformations (CVMs) and affects 0.5–1/1000 live births. The copy number variant (CNV) at chromosome 16p11.2 has been implicated in CVMs and recent studies identified a compound heterozygosity of 16p11.2 microdeletion and *TBX6* variant/haplotype causing CS in multiple cohorts, which explains about 5–10% of the affected cases. Here, we studied the genetic etiology of CS by analyzing CNVs in a cohort of 67 patients with congenital hemivertebrae and 125 family controls. We employed both candidate gene and family-based approaches to filter CNVs called from whole exome sequencing data. This identified 12 CNVs in four scoliosis-associated genes (*TBX6*, *NOTCH2*, *DSCAM*, and *SNTG1*) as well as eight recessive and 64 novel rare CNVs in 15 additional genes. Some candidates, such as *DHX40*, *NBPF20*, *RASA2*, and *MYSM1*, have been found to be associated with syndromes with scoliosis or implicated in bone/spine development. In particular, the *MYSM1* mutant mouse showed spinal deformities. Our findings suggest that, in addition to the 16p11.2 microdeletion, other CNVs are potentially important in predisposing to CS.

## 1. Introduction

Among all musculoskeletal disorders, scoliosis is one of the most common diseases, affecting around 3% of the world population, which can occur as an isolated defect or as a concomitant symptom in other diseases or syndromes [1]. Scoliosis is categorized into several main groups, including congenital scoliosis (CS), idiopathic scoliosis (IS), neuromuscular scoliosis, and degenerative scoliosis. CS, which usually has first onset at birth or shortly after birth, affects approximately 0.5–1 in 1000 live births [2,3,4,5]. Compared with IS, CS is generally more severe due to the high risk of progressive deformity and associated problems such as pulmonary compromise [6]. One of the most significant differences between CS and IS is that IS does not have an association with congenital vertebral malformation (CVM), whereas CVM is the major cause leading to CS. CVM can be classified into several subclasses, including failure of vertebral formation (e.g., hemivertebrae, wedged vertebrae), failure of vertebral segmentation (e.g., unilateral bar, block vertebrae), and mixed type. Of all CVMs, congenital hemivertebrae is the most common anomaly that causes CS [4,5].

During vertebral development, the paraxial mesoderm forms bilaterally paired blocks, named somites, along the anterior–posterior axis. The vertebral bodies are derived from somites formed in the presomitic mesoderm. This fundamental process is called somitogenesis. Once somitogenesis is disturbed, the resulting CVM may lead to spinal deformities. The most commonly accepted mechanism governing somitogenesis is the clock and wavefront model, which is controlled and coordinated by several key signaling pathways, such as Notch, Wnt, Fgf and retinoic acid signaling pathways [7,8]. Genetic studies of human patients with CVM have identified a variety of mutations in components of Notch signaling pathway (e.g., *NOTCH2*, *DLL3*, *MESP2*, *LFNG*, *HES7*, and *RIPPLY2*) and also in several key transcription factors essential for somitogenesis (e.g., *TBX6*, *TBXT*, and *SOX9*). Nevertheless, the genetic basis for majority of patients with CS still remains unclear [1,9].

Copy number variation (CNV) is a type of structural variation of genome. With the advancement of genome-wide analysis tools, it has been revealed that CNVs are widespread in the human genome and account for a large fraction of human genetic diversity [10]. CNVs have been, so far, implicated in many disease states including scoliosis. Although a number of CNVs were found to be associated with adolescent idiopathic scoliosis (AIS) [11,12], there have not been many reports about CS-associated CNVs. The 16p11.2 microdeletion was found to be associated with CS [13], and recent studies demonstrated that a compound inheritance of a *TBX6*-containing 16p11.2 microdeletion and a *TBX6* mutation or hypomorphic haplotype accounted for 5–10% of patients with CS in different populations [14,15,16,17]. Additional CNVs, including 10q24.31, 17p11.2, 20p11, 22q11.2, and a few other regions, were respectively reported in individual patients with CVMs [18,19]. Besides 16p11.2 microdeletion, it is unknown whether other CNVs are prevalent in CS.

Here, we analyzed CNVs in a Southern Chinese cohort of patients with congenital hemivertebrae. CNVs were called from whole-exome sequencing (WES) data of 67 cases and 125 family members (controls). We identified 12 rare CNVs in 4 known scoliosis-associated genes and eight recessive CNVs in three genes. We also found 64 novel, rare CNVs in 14 genes that occurred in multiple patients but are very rare in our control group and the general population, suggesting a potential role for genetic susceptibility in the development of CS.

## 2. Materials and Methods

### 2.1. Patient Recruitment

The patients studied in this project were recruited from the Duchess of Kent Children’s Hospital (DKCH), a tertiary scoliosis referral center in Hong Kong. The patients with CS were diagnosed by imaging such as plain standing whole-spine radiographs and computed tomography. A total of 67 patients with hemivertebrae were chosen for this study, of which 31 had single congenital hemivertebrae while 36 had multiple congenital hemivertebrae. Patients’ personal data and medical records were collected under ethical privacy guidelines and approval. Ethics was approved by the Institutional Review Board of the University of Hong Kong/Hospital Authority Hong Kong West Cluster (HKU/HA HKW IRB Ref # UW 15-216), and written informed consent was obtained from all participants and/or parents/siblings.

### 2.2. Control Cohort

The control cohort studied in this project consisted of 125 participating family members of the recruited patients with CS. Only unaffected parents and siblings (without CS) were included. Accordingly, 58 out of 67 patients had family member(s) participating in this study, including 2 quintets, 14 quartets, 33 trios, and 9 duos. 

### 2.3. Genomic DNA Extraction

Genomic DNAs were extracted from peripheral blood samples of 67 patients and 125 of their family members using Invitrogen^TM^ ChargeSwitch gDNA Serum Kit. The purified genomic DNA was quantified by NanoDrop.

### 2.4. Whole-Exome Sequencing (WES) and Copy Number Variations (CNVs) Calling

WES was performed for all recruited patients with congenital hemivertebrae and participating family members by Novogene Co, Ltd. (Hong Kong, China), using the Agilent SureSelect Human All Exon Kit on the Illumina sequencing platform. The WES data were processed as described previously [20]. The raw sequence data were first analyzed by fastp for quality control and filtering [21]. After filtering, the Q20 base of most samples was greater than 95%, and the Q30 base was greater than 90%. The sequence reads were mapped to the reference genome (GRCh37/hg19) by Burrows-Wheller Aligner v0.7.17 (BWA-MEM) [22] and further processed using SAMtools v1.10 to sort and index aligned reads [23]. The sam format files generated by BWA was converted to bam format files by SAMtools. CNVs were called from bam files with ExomeDepth v1.1.15, which is an R package based on a read depth algorithm [24]. ExomeDepth uses a robust statistical model to build an optimized reference set in maximizing the CNVs detection power. In this study, four healthy control family members (CS59A, CS71A, CS71B, and CS81A) were selected to generate the reference set.

### 2.5. CNVs Filtering

Several criteria were used to filter CNVs: (i) Bayes factor (BF) values were calculated for each variant. BF equals to the log10 likelihood ratio of the alternative hypothesis (i.e., there is a CNV) over the null hypothesis (i.e., there is no CNV). BF = log10 (alternative hypothesis/null hypothesis). BF value greater than 1 was regarded as a strong supporting evidence of CNV. CNVs with BF values smaller than 1 were excluded. (ii) As ExomeDepth cannot detect small size CNVs accurately, CNVs with size smaller than 100 bp were excluded. (iii) Because CNVs with high allele frequency in the general population are likely benign and less susceptible, the CNVs with the allele frequency greater than 0.01 were excluded (a minimum sample size of 100 is required). Database of Genomic Variants (DGV) and Genome Aggregation Database (gnomAD) were used. If available, the allele frequency in East Asian population was also checked. As different CNVs often overlap and have no clear boundaries, this filtration was conducted in a gene-based manner. If there were multiple CNVs covering the same gene, the maximum allele frequency was used for filtering. (iv) In a gene-based manner, the number of CNV recurrence was counted in patients and controls.

### 2.6. Real-Time Quantitative PCR (qPCR)

Real-time qPCR was performed to validate some of the detected CNVs. Briefly, ROX Reference Dye (0.4 μL, 50X), forward and reverse primers (0.4 μL each, 10 μM), TB Green Premix Ex Taq (10 μL, 2X, Tli RNaseH Plus, Takara), patients’ genomic DNA (0.5 μL, 10 ng/μL), and sterile ddH_2_O (8.3 μL) were mixed for qPCR, which was performed using Applied Biosystem^TM^ StepOnePlus^TM^ Real-Time PCR System. A locus outside of the detected CNV region of *NOTCH2*, *DSCAM* and *SNTG1* was used as reference locus (P1). P1 is near the region of chromosome 16p11.2 and previously used as a reference site to detect 16p11.2/TBX6 deletion [14,17]. Each sample was analyzed in triplicate. Quantities of the copy numbers of specific locus were determined by the delta Ct method. The 2^−ΔΔCT^ method was used to analyze the relative changes. The qPCR primer sequences: *NOTCH2*-F: 5′- AGGAGGCGACCGAGAAGATG-3′; *NOTCH2*-R: 5′-CGATACTCACCATGCGCG-GG-3′; *DSCAM*-F: 5′-AGCGAACGTTCCTATCGCTT-3′; *DSCAM*-R: 5′-TTTCACTTATGCGCCCTGGG-3′; *SNTG1*-F: 5′-GTCTACATGGGCTGGTGTGA-3′; *SNTG1*-R: 5′-CTGGAGGTGCCAGAAACTTG-3′; P1-F: 5′-GGGGAAGGAACTTACATGAC-3′; P1-R: 5′-TCGTGTTTCCCTGTTGTACC-3′.

## 3. Results

### 3.1. CS Cohort and WES

In our cohort, we recruited a total of 92 patients with CS, in which vertebral malformations, such as hemivertebrae, unilateral bar, or block vertebrae, were identified. This operational definition thus excluded other types of scoliosis such as AIS. Because hemivertebrae is the most common type of vertebral malformation in CS and has the greatest potential for rapid progression (5–10 degrees/year) [25], 67 patients with congenital hemivertebrae were first selected. Further, 125 healthy family members of 58 patients were enrolled for this study, including parents and siblings from two quintets, 14 quartets, 33 trios, and nine duos. WES was performed for all 67 patients and 125 participating family members (controls). The contaminating sequencing adaptors and low-quality reads were first removed and the filtered reads were then aligned to the reference human genome (GRCh37/hg19), sorted and indexed.

### 3.2. CNV Calling

**Figure 1:**
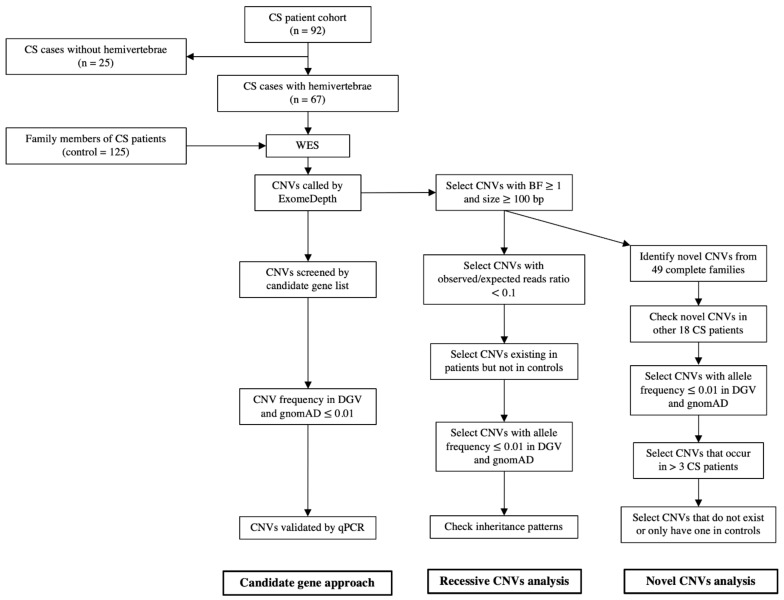
The workflow of CNV analysis. This strategy detected CNVs in several candidate genes and identified recessive and novel rare CNVs enriched in patients with CS.

CNVs were called from the sequence reads with the read-depth analysis tool ExomeDepth, which has high sensitivity and specificity at the exon level [24,26]. Four healthy parents who were not carriers of 16p11.2 microdeletion but whose children have been previously diagnosed with TBX6 compound heterozygosity [17] were selected to generate the reference set for ExomeDepth analysis. After CNV calling of the 67 patients with CS, a total of 15,671 CNVs were detected. On average, each patient carries around 234 CNVs. By counting repeatedly occurring CNVs among different cases, there were 6084 distinct CNVs. This strategy successfully identified *TBX6*-containing 16p11.2 microdeletion in four patients as previously reported [17]. For the control group, a total of 27,116 CNVs were detected from 125 family control members. On average, each control carried around 217 CNVs. By counting repeatedly occurring CNVs among different controls, there were 7171 distinct CNVs. Although more CNVs were detected in a few individuals (six patients and four controls), there was no significant difference between the patient group and the control group (Supplementary Figure S1). The average CNV numbers in patients and controls were similar to the previous report [24]. Afterwards, we analyzed all CNVs by employing both a candidate gene approach and family-based filtering and prioritization strategies. A workflow is shown in Figure 1.

### 3.3. CNVs in Candidate Genes

To identify CNVs associated with CS, we firstly used the candidate gene approach, and focused on CNVs that contained genes known to be involved in scoliosis or somitogenesis. After checking allele frequencies of CNVs in the Database of Genomic Variants (DGV) and the Genome Aggregation Database (gnomAD), a total of 12 rare CNVs that influence four candidate genes were found in 12 patients, including known *TBX6*-containing 16p11.2 heterozygous deletion in four cases [17]. We also identified two rare CNVs that contained *NOTCH2*, a key component in the Notch signaling pathway, in two patients, and six rare CNVs in AIS-associated genes, *DSCAM* [27] and *SNTG1* [28,29], in six patients (Table 1). We then checked these CNVs in their available family members and found that they are either *novel* mutations (*NOTCH2* in CS043, *DSCAM* in CS018 and CS036, and *SNTG1* in CS048) or paternally inherited (*DSCAM* in CS050) (Table 1). We were unable to determine the inheritance patterns of other patients (*NOTCH2* in CS033, *DSCAM* in CS053 and CS064) due to the lack of family members.

Among the identified rare CNVs, the TBX6-containing chromosome 16p11.2 microdeletion had been previously validated [17]. Here, we further examined CNVs that contained *NOTCH2*, *DSCAM* or *SNTG1* genes. Indeed, qPCR analysis detected heterozygous deletions within the *NOTCH2*, *DSCAM* and *SNTG1* loci (Supplementary Figure S2), indicating the reliability of CNVs called from WES data by ExomeDepth.

### 3.4. Recessive CNVs in Patients with CS

We then searched for homozygous CNVs (observed/expected reads ratio < 0.1) in 67 patients and 125 controls. After excluding the homozygous CNVs that existed in both patients and controls, we identified unique homozygous CNVs in eight patients with CS. The heterozygous deletions of these loci are rare in DGV or gnomAD database (Table 2). Considering that homozygous CNVs might be inherited from parents, we further checked their inheritance pattern and found that they were either *novel* mutations or unknown due to lack of parents’ data. These recessive CNVs contained three genes, *NBPF20* (Neuroblastoma Breakpoint Family Member 20), *FAM138C* (Family with Sequence Similarity 138 Member C), and *DHX40* (DEAH-Box Helicase 40). Interestingly, the *DHX40*-containing homozygous CNVs were detected in six patients but was not reported in DGV or gnomAD. *DHX40*-containing heterozygous CNVs are also very rare (Table 2). *FMA138C* is an RNA gene and *NBPF20* is a member of NBPF family characterized by tandemly repeats of DUF1220 domain, but their functions are unclear. *DHX40* encodes a member of the DExD/H-box RNA helicase superfamily that catalyzes the unwinding of double-stranded RNA and has an essential role in RNA metabolism [30].

### 3.5. Novel CNVs in Patients with CS 

We also sought to identify CS-associated novel CNVs and first analyzed the data from 49 complete families (two quintets, 14 quartets or 33 trios). The detected novel CNVs were then checked in the other 18 patients (nine singlets and nine duos). Eventually, we identified 64 CNVs in 14 genes that occurred in more than three patients but did not exist or was very rare (<1%) in family control group. Those with high CNV allele frequency (>1%) in the general population were also filtered out. This strategy successfully identified the known *TBX6*-containing CNVs in four patients [17] and *DHX40*-containing homozygous CNVs in six patients. Interestingly, we also found there are four additional heterozygous *DHX40* CNVs (Table 3 and Supplementary Table S1). Most of the identified novel CNVs were heterozygous loss, and one was gain of one copy. Our CNV shortlist includes genes involved in ubiquitination (*NAE1*, *MYSM1*), enzymatic activities (*MME*, *PHKB*), ion/small molecule transportation (*SCN7A*, *ABCA6*), meiosis (*MNS1*, *SPO11*), spermatogenesis (*GMCL1*), GTPase activity (*RASA2*), TNF signaling (*NSMAF*), or with unknown function (*LRRC40*).

## 4. Discussion

CS is a genetically heterogeneous disorder with evidence for multiple causative genes. However, the genetic causes of the majority of patients still remain unknown. As most cases of CS are of sporadic etiology, CNVs may have greater influence than single nucleotide variations (SNVs) [31]. This was well exemplified by the *TBX6*-containing 16p11.2 microdeletion in previous CS studies [14,15,16,17]. Here, we systematically investigated CNVs in a cohort of patients with congenital hemivertebrae and their family controls. We identified the well-known CNVs at chromosome 16p11.2, as well as a number of new CNVs that are potentially associated with CS. Haploinsufficiency of Notch signaling pathway has been demonstrated to cause CS [32] and mutations in *NOTCH2* caused Alagille syndrome and Hajdu–Cheney syndrome, both of which showed abnormal curvature of the spine [33,34]. In our study, we found one short and one long CNVs at *NOTCH2* locus in two patients, spanning one and four exons of *NOTCH2*, respectively. As no significant coding SNV could be detected in *NOTCH2* of these two patients, it is unclear whether heterozygous loss of *NOTCH2* is sufficient to cause CS or other non-coding *NOTCH2* SNVs or environmental factors [32] may contribute. Interestingly, CNVs in two AIS-associated genes, *DSCAM* [27] and *SNTG1* [28,29], were found in six patients, suggesting CS and AIS may be genetically related to each other.

An intriguing finding in our analysis is the identification of CNVs spanning various exons of *DHX40* in ten patients, including six homozygous and four heterozygous CNVs. Most of them are novel mutations. *DHX40* belongs to the conserved DExD/H-box RNA helicase family, which facilitates the ATP-dependent unwinding of RNA secondary structures [30]. However, the biological functions of each member remained poorly understood. Interestingly, the *DHX40* mutant mice were described to exhibit abnormal bone structure and bone mineralization (Mouse Genome Informatics, MGI: 1914737), indicating a role of *DHX40* in bone development. The mutant of its family member *DHX35* was described to have abnormal vertebrae morphology and scoliosis in mice (MGI: 1918965). Patients carrying *DHX37* mutations showed developmental delay and intellectual disability as well as vertebral anomalies [30]. These observations in the mouse and human might suggest a potential link between DHX family members and CVM. 

Although the function of *NBPF20* is unknown, it locates at chromosome 1q21.1, which microdeletion is associated with a variety of phenotypes including skeletal malformations such as scoliosis [35,36]. This region also contains other NBPF family members, such as *NBPF10*, whose genetic variants were implicated in Mayer-Rokitansky-Küster-Hauser (MRKH) syndrome (OMIM # 277000) [37], a disease associated with CS [38].

Among the candidate genes of the identified novel CNVs, *RASA2* (RAS P21 Protein Activator 2) and *MYSM1* (Myb-Like, SWIRM, and MPN domains 1) are of particular interest. *RASA2* encodes a GAP (GTPase-activating protein) protein and functions as a suppressor of RAS by promoting its intrinsic GTPase activity. Rare variants in *RASA2* have been found associated with Noonan syndrome [39]. As scoliosis occurs frequently in Noonan syndrome [40], *RASA2* is a potential candidate gene for CS. It would be interesting to investigate the *RASA2* mutant mouse phenotype. MYSM1 is a deubiquitinase reported to be essential for bone formation [41] and its mutant mice have truncated and kinky tails [42,43,44], which are often associated with vertebral malformations [45]. Indeed, an X-ray from the International Mouse Phenotyping Consortium (MGI: 2444584) exhibited a spinal deformity in the *MYSM1* mutant mouse (Supplementary Figure S3), indicating a potential role of *MYSM1* in spinal development and predisposition to CS. Further detailed phenotypic analysis of mutant animals is needed to validate its pathogenicity in CS.

Although there are a few reports of heterozygous point mutations in CS patients [17,46,47,48], the dominant negative effect was only demonstrated by a novel *TBXT* mutation [17]. Considering the low familial recurrence rate in CS, recessive or compound heterozygous mutations are more likely to be the major cause of CS. In this regard, heterozygous CNVs are not sufficient to induce CS. Their pathogenicity may be explained by the following genetic models. First, in our cases, the patients carrying heterozygous CNVs may have additional risk variant or haplotype on the other allele. This possibility has been well exemplified by the 16p11.2/TBX6 mutations and haplotype. However, further analysis of risk variant/haplotype in our study is severely limited by our dataset from WES as they may reside in non-coding regions that regulate gene transcription. We did not detect significant deleterious mutations in the coding regions of these genes. Second, additional mutations in other relevant genes may increase the risk of CS (polygenic model). Other possibilities include environmental contributions and novel mutations in somatic tissues. Environmental factors, such as short-term gestational hypoxia, have been found to cause CS in combination with haploinsufficiency of Notch signaling pathway genes [32]. On the other hand, somatic mutations may serve as the “second hit” in addition to the heterozygous germline CNV mutations (first hit). This genetic model has been well demonstrated in other diseases as well as dystrophic scoliosis caused by *NF1* [49,50,51,52]. Testing the above models will require whole genome sequencing, more comprehensive data analysis, and the isolation of malformed vertebral tissues in future studies.

## 5. Conclusions

In this study, we investigated the genetic basis of CS by analyzing CNVs in a cohort of CS families. Based on the candidate gene approach and family-based filtering of CNVs, we identified both known CS-associated genes and a set of new susceptibility genes, some of which (e.g., *DHX40*, *RASA2*, and *MYSM1*) warrant further investigations in larger cohorts as well as functional characterization. Given the well-defined example of the *TBX6* compound inheritance and the complex genetic nature of CS, future studies examining the combined effects of SNVs and CNVs and somatic tissues may help better decipher the genetic etiology and heterogeneity of CS.

## Figures and Tables

**Table 1:** CNVs found with candidate gene approach (N.D., not determined; N.A., not applied).

Gene	Patient	Type	Chr	Start	End	Size (bp)	Bayes Factor	Reads Ratio (Observed/ Expected)	Exons Annotation (hg19) (Gene_exon)	Inheritance Pattern	Highest Frequency in DGV (Sample Size >100)	gnomAD_ Structural_Variants Frequency (Heterozygous Loss)
*NOTCH2*	CS033	deletion	1	120,611,949	120,612,020	71	4.62	0.657	NOTCH2_1	N.D.	0.0037	0.00037 (0 in East Asia)
	CS043	deletion	1	120,539,621	120,612,020	72,399	8.46	0.722	NOTCH2_1-4	De novo	0.0037	0.00037 (0 in East Aisa)
*DSCAM*	CS018	deletion	21	41,452,080	41,452,267	187	4.65	0.429	DSCAM_25	De novo	0.00049	N.A.
	CS036	deletion	21	41,452,080	41,452,267	187	6.02	0.415	DSCAM_25	De novo	0.00049	N.A.
	CS050	deletion	21	41,452,080	41,452,267	187	5.8	0.468	DSCAM_25	Paternal	0.00049	N.A.
	CS053	deletion	21	41,452,080	41,452,267	187	5.45	0.494	DSCAM_25	N.D.	0.00049	N.A.
	CS064	deletion	21	41,452,080	41,452,267	187	6.1	0.463	DSCAM_25	N.D.	0.00049	N.A.
*SNTG1*	CS048	deletion	8	51,503,440	51,571,223	67,783	6.37	0.43	SNTG1_13-15	De novo	0.0002	0.000046 (0 in East Asia)
*TBX6*	CS059	deletion	16	29,674,601	30,199,897	525,296	644	0.555	SPN_2,AC009133.19_2-3,QPRT_1-4,C16orf54_2,ZG16_2-4,KIF22_1-13,MAZ_1-5,PRRT2_2-3,PAGR1_1-3,CTD-2574D22.6_1-2,MVP_2-15,CDIPT_6-2,SEZ6L2_16-1,ASPHD1_1-3,KCTD13_6-1,TMEM219_1-4,TAOK2_2-16,HIRIP3_7-1,INO80E_1-7,DOC2A_11-2,C16orf92_2-3,FAM57B_5-1,ALDOA_8-16,PPP4C_2-9,**TBX6_9-2**,YPEL3_4-1,GDPD3_10-1,MAPK3_8-1,CORO1A_2-3,CORO1A_4-10	N.D.	0.0005	0.0001462 (0 in East Asia)
	CS071	deletion	16	29,495,011	30,218,221	723,210	754	0.572	NPIPL3_3-1,SPN_2,AC009133.19_2-3,QPRT_1-4,C16orf54_2,ZG16_3-4,KIF22_2-12,MAZ_1-5,PRRT2_2-3,PAGR1_1-3,CTD-2574D22.6_1-2,MVP_2-15,CDIPT_6-2,SEZ6L2_16-1,ASPHD1_1-3,KCTD13_6-1,TMEM219_1-4,TAOK2_2-16,HIRIP3_7-2,INO80E_1-7,DOC2A_11-2,C16orf92_2-3,FAM57B_5-1,ALDOA_8-16,PPP4C_2-9,**TBX6_9-2**,YPEL3_4-1,GDPD3_10-1,MAPK3_8-1,CORO1A_2-11,BOLA2B_3-1,SLX1A_1-5,SULT1A3_3-9,RP11-347C12.3_5-2	De novo	0.0005	0.0001462 (0 in East Asia)
	CS078	deletion	16	29,498,516	30,199,897	701,381	690	0.578	NPIPL3_1,SPN_2,AC009133.19_2-3,QPRT_1-4,C16orf54_2,ZG16_3-4,KIF22_2-12,MAZ_1-5,PRRT2_2-3,PAGR1_1-3,CTD-2574D22.6_1-2, MVP_2-15,CDIPT_6-2,SEZ6L2_16-1,ASPHD1_1-3,KCTD13_6-1,TMEM219_1-4,TAOK2_2-16,HIRIP3_7-2,INO80E_1-7,DOC2A_11-2,C16orf92_2-3,FAM57B_5-1,ALDOA_8-16,PPP4C_2-9,**TBX6_9-2**,YPEL3_4-1,GDPD3_10-1,MAPK3_8-1,CORO1A_2-10	N.D.	0.0005	0.0001462 (0 in East Asia)
	CS081	deletion	16	29,498,516	30,199,897	701,381	645	0.558	NPIPL3_1,SPN_2,AC009133.19_2-3,QPRT_1-4,C16orf54_2,ZG16_3-4,KIF22_2-12,MAZ_1-5,PRRT2_2-3,PAGR1_1-3,CTD-2574D22.6_1-2, MVP_2-15,CDIPT_6-2,SEZ6L2_16-1,ASPHD1_1-3,KCTD13_6-1,TMEM219_1-4,TAOK2_2-16,HIRIP3_7-2,INO80E_1-7,DOC2A_11-2,C16orf92_2-3,FAM57B_5-1,ALDOA_8-16,PPP4C_2-9,**TBX6_9-2**,YPEL3_4-1,GDPD3_10-1,MAPK3_8-1,CORO1A_2-10	N.D.	0.0005	0.0001462 (0 in East Asia)

**Table 2:** Recessive CNVs unique in patients with CS (N.D., not determined; N.A., not applied).

Gene	Patient	Type	Chr	Start	End	Size (bp)	Bayes Factor	Reads Ratio (Observed/Expected)	Exons Annotation (hg19) (Gene_exon)	Inheritance Pattern	Highest Frequency in DGV (Sample Size > 100)	gnomAD_ Structural Variants Frequency (Heterozygous Loss)
*NBPF20*	CS047	deletion	1	148,261,458	148,262,366	908	5.27	0.04	NBPF20_98-99	De novo	0	0 (0 in East Asia)
*FAM138C*	CS048	deletion	9	35,061	35,519	458	6.51	0	FAM138C_1-2	* De novo	0.0074	N.A.
*DHX40*	CS004	deletion	17	57,656,834	57,657,240	406	5.38	0	DHX40_9-10	N.D.	0.0000922	0.0025 (0.008152 in East Asia)
	CS035						4.91			N.D.		
	CS043						6			* De novo		
	CS050						7.23			* De novo		
	CS053						7.02			N.D.		
	CS057						6.29			De novo		

* The CNV is also not present in the healthy siblings.

**Table 3:** Novel CNVs enriched in patients with CS (N.A., not applied).

Gene	Type	Chr	Size (bp)	Count in 67 Patients	Count in 125 Controls	Highest Frequency in DGV (Sample Size > 100)	gnomad_ Structural_Variants Frequency (Heterozygous Loss)	gnomad_East Asia_Structural_Variants Frequency (Heterozygous Loss)
*LRRC40*	deletion	1	30,080–383,938	4	0	0.009556907	0.0000461	0.000
*SCN7A*	deletion	2	544–11,914	4	0	0.002257336	0.0000461	0.000
*MME*	deletion	3	279–55,232	4	0	0.000798722	0.0000462	0.000
*NAE1*	deletion	16	6724–71,803	4	0	N.A.^b^	0	0.000
*TBX6*	deletion	16	525,296–723,210	4	0	0.0005	0.0001462	0.000
*DHX40*	deletion	17	107–2354	10 ^a^	1	0.0000922	0.0025	0.008152
*GMCL1*	deletion	2	11,694–24,032	5	1	0.001303781	0.0000479	0.000
*MYSM1*	deletion	1	190–10,053	4	1	0.0000922	0.0000461	0.0004139
*RASA2*	deletion	3	2892–100,149	4	1	0.00086881	0	0.000
*NSMAF*	deletion	8	203–12,325	4	1	0.001145475	0	0.000
*MNS1*	deletion	15	52,610–323,156	4	1	0.00518807	0.0000922	0.000
*PHKB*	deletion	16	7697–99,254	4	1	0.001198083	0.0000481	0.000
*SPO11*	deletion	20	730–33,608	4	1	0.00064226	0	0.000
*ABCA6*	duplication	17	560–13,580	4	1	0.0009219	0	0.000

^a^ DHX40 has 6 homozygous (listed in Table 2) and 4 heterozygous CNVs. ^b^ No CNV with sample size more than 100 is found within the *NAE1* locus. Note: Detailed information of these novel CNVs is shown in Table S1.

## Data Availability

Data available on request due to restrictions.

## Supplementary Materials

The following are available online at https://www.mdpi.com/article/10.3390/genes12081213/s1, Figure S1: Total number CNVs in each patient and family control, Figure S2: Quantitative PCR analysis for validating heterozygous deletion, Figure S3: The X-ray of *MYSM1* mutant mouse from IMPC. Table S1: Novel CNVs enriched in CS patients.
