# Supplementary material for: Identification of Copy Number Variants in a Southern Chinese Cohort of Patients with Congenital Scoliosis
Source: Genes (Basel). 2021 Aug 5;12(8):1213. doi: 10.3390/genes12081213 (PMC8391542; doi:10.3390/genes12081213)
Supplement: Supplementary file 1 [file genes-12-01213-s001.zip › Supplementary Figures.pdf]

Supplementary Figure 1

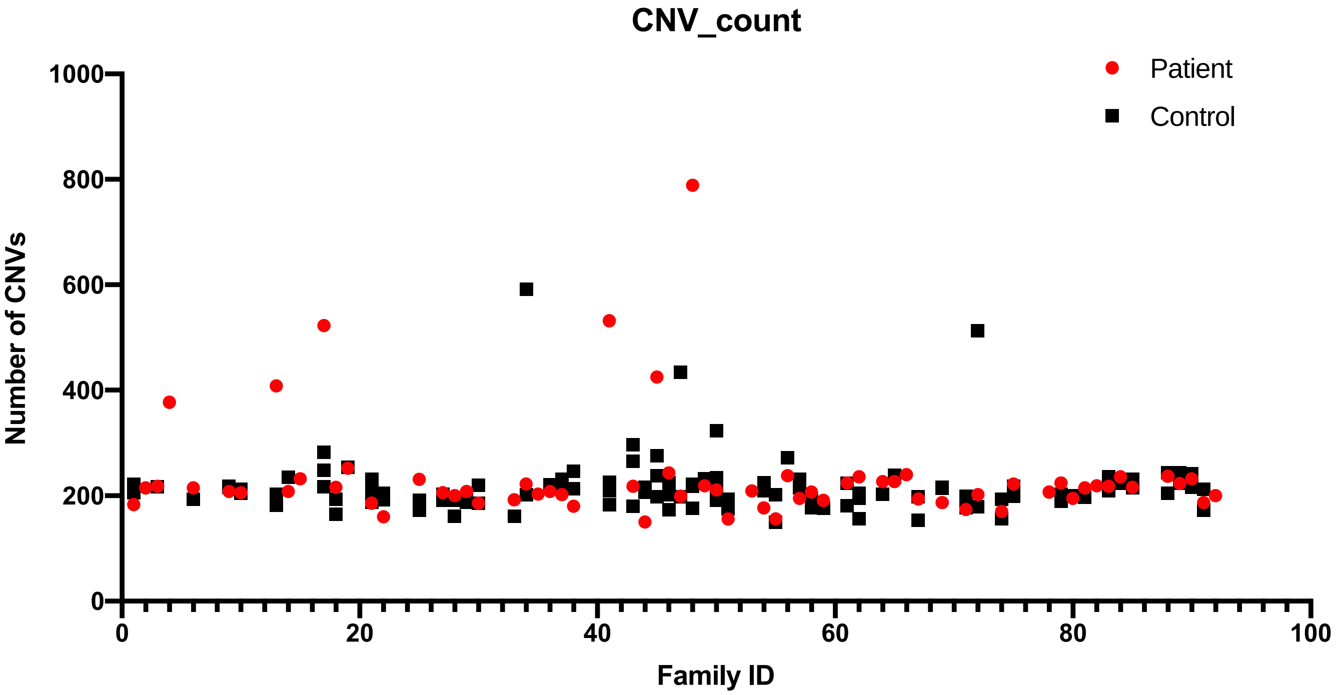

Figure S1. Total number of CNVs called from whole exome sequencing data by ExomeDepth in each patient and family control. There is no statistically significant difference between patient and control groups (student *t* test, p=0.207).

Supplementary Figure 2

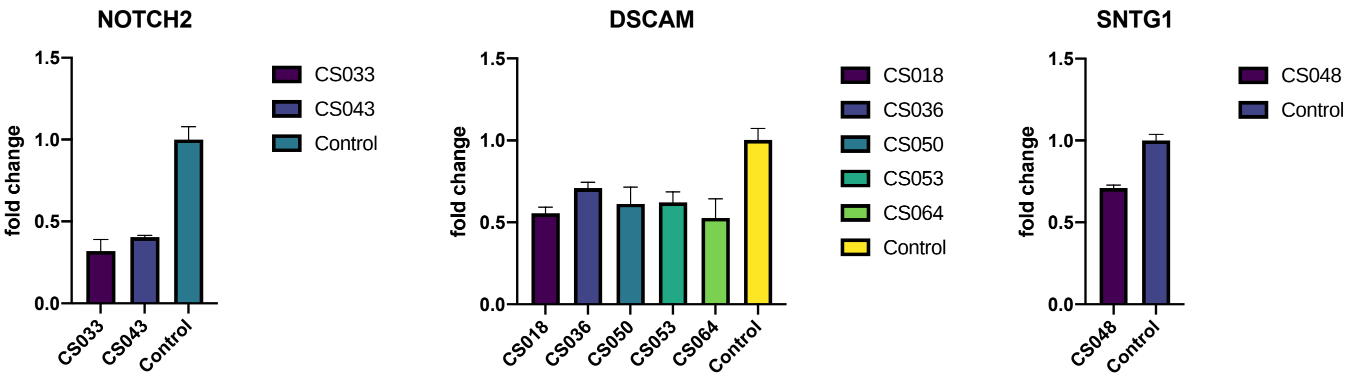

Figure S2. Quantitative PCR analysis for validating the heterozygous deletion on *NOTCH2*, *DSCAM*, and *SNTG1* loci. The locus (P1) outside the deletion region was used as a reference site. Control samples were selected from family members with normal copy number on the these loci.

Supplementary Figure 3

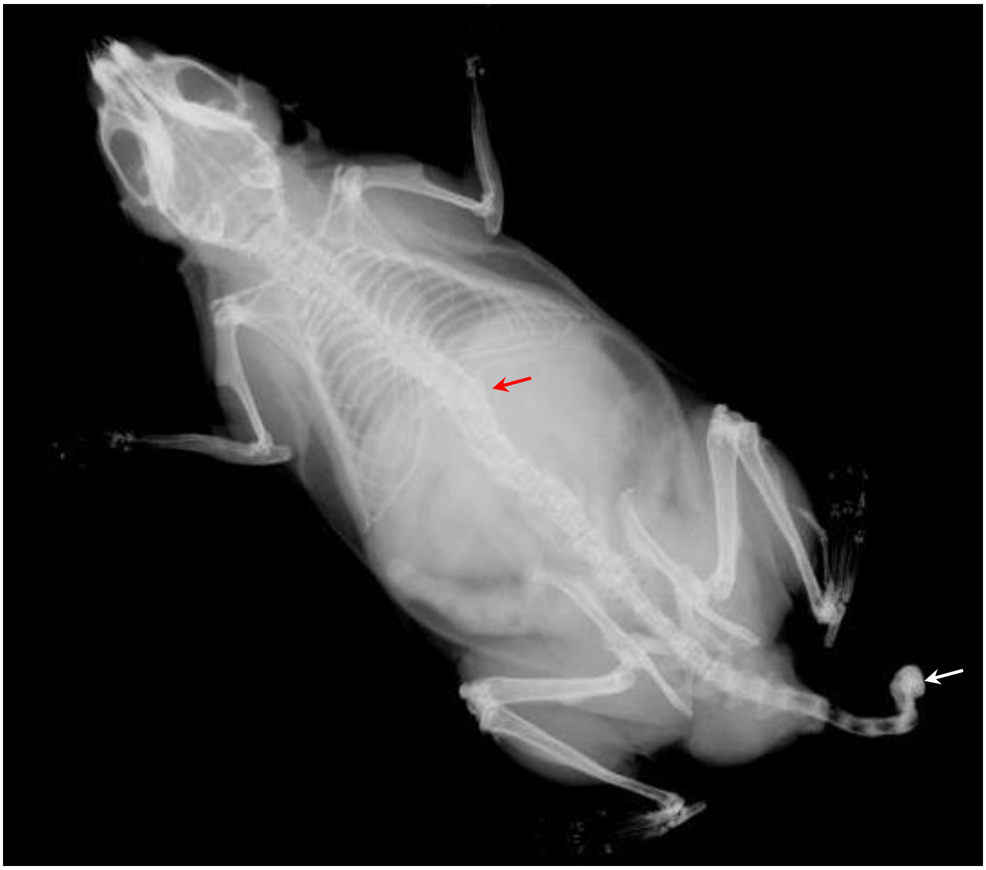

Figure S3. The X-ray of *Mysm1* homozygous mutant mouse (*Mysm1$^{tm1a(KOMP)Wtsi}$*) obtained from International Mouse Phenotyping Consortium (IMPC) website (https://www.mousephenotype.org/data/genes/MGI:2444584). The red arrow points to the place of vertebral malformation and the white arrow points to the truncated and kinky tail.
